# Bicycle handlebar injury in a child resulting in complex liver laceration with massive bleeding and bile leakage: A case report

**DOI:** 10.1016/j.ijscr.2020.06.049

**Published:** 2020-06-12

**Authors:** Jan Grosek, Žan Čebron, Jurij Janež, Aleš Tomažič

**Affiliations:** a Department of Abdominal Surgery, University Medical Centre Ljubljana, Zaloska 7, 1000, Ljubljana, Slovenia; b Medical Faculty, University of Ljubljana, Vrazov trg 2, 1000, Ljubljana, Slovenia

**Keywords:** HBI, handlebar injury, HPB, hepato-pancreato-biliary, ICU, intensive care unit, MAP, mean arterial pressure, US, ultrasound, FAST, focused assessment with sonography for trauma, PDS, polydioxanone suture, AAST, American Association for the Surgery of Trauma, ATLS, advanced trauma life support, AST, aspartate aminotransferase, ALT, alanine aminotransferase, Bicycle accidents, Handlebar injury, Liver laceration, Pediatric trauma, Case report

## Abstract

**Introduction:**

Bicycle accidents are a significant cause of traumatic morbidity in the paediatric population. The handlebar injuries are usually isolated and remain a major source of bicycle related morbidity. We present a case of severe liver laceration with left hepatic duct transection caused by handlebar trauma in a 13-year-old boy.

**Case presentation:**

An otherwise healthy 13-year-old Caucasian male patient was rushed to the hospital following a blunt abdominal trauma from a bicycle handlebar. An ultrasound revealed extensive free intraperitoneal fluid with accompanying features of hemodynamic instability. Operative findings included massive haemoperitoneum, a deep laceration almost separating left and right liver lobes, and a near-complete interruption of the left hepatic duct. Interestingly, the vascular anatomy of the left liver lobe was preserved. After haemostasis was established a duct-to-duct anastomosis of the ruptured left hepatic duct was performed. A T-tube biliary drainage was inserted, and intraoperative cholangiography showed no extraluminal spillage of contrast. During the recovery phase, cholangiography was performed several times revealing leakage from left hepatic duct. The leakage was managed conservatively and finally stopped.

**Discussion:**

Management of blunt hepatic injuries has remarkably changed from mandatory operation to mostly nonoperative interventions with selective operative management. Hemodynamic instability remains the main reason for exploratory laparotomy. Near-isolated laceration of main hepatic ducts is an extremely rare finding, and immediate reconstruction is the best option if an experienced surgeon is available.

**Conclusion:**

Due to limited evidence, we recommend consultation with an experienced HPB surgeon on a case-by-case basis for every paediatric biliary injury.

## Highlights

• Bicycle injuries represent a significant cause of traumatic morbidity.
• A case of HBI causing severe liver laceration is presented.
• Near-isolated laceration of main hepatic ducts is an extremely rare surgical finding.
• Paediatric biliary injury should be managed by experienced HPB surgeon.

## 1 Introduction

Bicycle injuries represent a significant cause of traumatic morbidity among the paediatric population [[1], [2], [3]]. Besides high-speed bicycle traumas causing multiorgan injuries, isolated handlebar injuries (HBI) remain a major source of bicycle-related morbidity, with nearly one third of cases requiring surgical intervention [1,3,4]. The spectrum of injuries varies widely from solid organ injury to traumatic abdominal wall hernias and bowel injuries [5]. Despite minimal or absent visible external signs on the abdominal wall, which is the most frequent site of impact, bicycle HBI should be treated with great care [2,[6], [7], [8]].

In the present report, we describe the clinical presentation, management and outcome of HBI causing severe liver laceration with haemorrhage and left hepatic duct injury in a young boy. The case report is reported in line with the SCARE criteria [9].

## 2 Case presentation

A 13-year-old Caucasian male patient presented to our surgical emergency department with complaints of severe diffuse abdominal pain and distension. The patient was brought to the hospital by helicopter about 2 h after sustaining a direct blow to the upper right quadrant of his abdomen from a bicycle handlebar. At the scene of the accident, all resuscitation measures were given in line with the recommendations of ATLS.

The patient was conscious and well-oriented. During the helicopter transportation, he was noted to be pale with blood pressure around 90/60 mmHg, a heart rate of 110–120 beats/min and haemoglobin 85 g/L. Despite a slightly higher blood pressure (115/80 mmHg) following fluid resuscitation upon admission, he remained pale and tachycardic (120 beats/min). Abdominal examination was remarkable for significant distension and diffuse tenderness, but no visible external injuries on the abdominal wall.

Chest x-ray showed no signs for rib injury, pneumothorax or any other expected pathology. FAST scan was performed, showing diffuse echogenic free fluid intraperitoneally with the most probable cause being hemoperitoneum. There were some radiologic signs of contusion of the upper part of left liver lobe, but no signs of splenic injury. Due to the extensive intraperitoneal free fluid seen on FAST and the persisting features of haemodynamic instability (tachycardia, pallor), a decision for immediate exploratory laparotomy and omitting abdominal CT was made. Performing CT imaging would have postponed the operation and might have influenced the outcome.

An emergency upper median laparotomy was performed under general anaesthesia to determine the exact nature of the abdominal injury. Due to the hematoperitoneum, our operative procedure was in conformity with damage control surgery and tamponade of all four abdominal quadrants was performed.

**Fig. 1:**
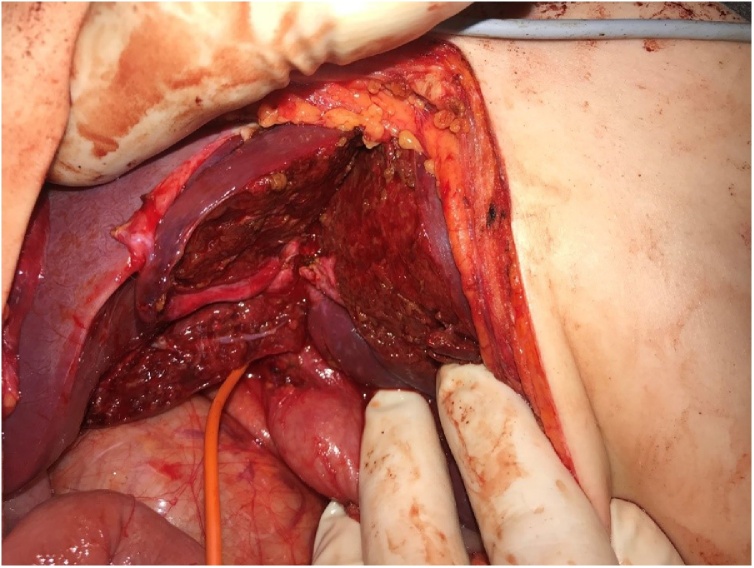
**Liver laceration with T- tube inserted into the common bile duct**.

During the exploration, a deep laceration almost separating left and right liver lobes was found with evident bleeding from the laceration (grade II-III according to AAST liver injury scale). Firstly, with the intention to stop the bleeding, the Pringle manoeuvre was performed, and venous bleeding was stopped with several sutures. Thereafter, a meticulous examination of the laceration was done, and an injury of the left hepatic duct was observed. The left hepatic duct was interrupted almost completely, with less than 10% of the circumference preserved. However, the portal and arterial vessels for the left lobe were mainly preserved. A duct-to-duct anastomosis of the injured left hepatic duct was performed with 6.0 PDS intermittent sutures. Subsequently, cholecystectomy, insertion of biliary T-tube and intraoperative cholangiography were performed (Fig. 1). Intraoperative cholangiography showed no extraluminal spillage of contrast. At the end of the operation, hemostatic absorbable wraps Surgicel® were inserted into the liver laceration. Intraoperatively, the patient received 3 units each of fresh frozen plasma and concentrated erythrocytes, as well as crystalloid fluids and tranexamic acid.

After operation he was admitted to the paediatric ICU under analgosedation and endotracheal intubation. Immediate post-op laboratory data were as follows: AST 7.02 μkat/L, ALT 7.81 μkat/L, total bilirubin 22 μmol/L, direct bilirubin 10 μmol/L, haemoglobin 107 g/L, erythrocytes 3.76 × 10^12^, leukocytes 10.4 × 10^9^ and CRP below 5 mg/L. Sedation and vasoactive support were discontinued within the first postoperative day.

On the second postoperative day, an US scan was performed, showing minimal free fluid in the peritoneal cavity under the liver. The bile ducts diameters were within normal range.

**Fig. 2:**
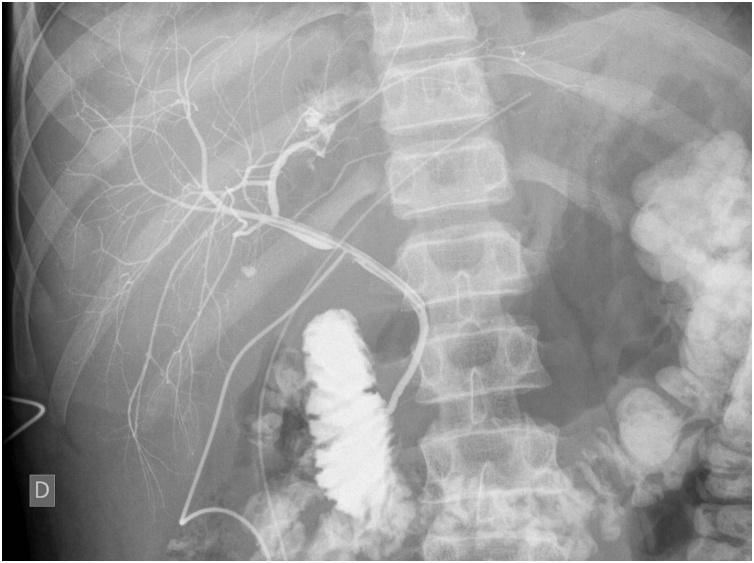
**The cholangiography through the T-drain on the 5th postoperative day**. Leakage from left hepatic duct is seen. There is a small collection of contrast in the right biliary tree. Normal opacification of the common hepatic duct and common bile duct is noted.

On the fifth postoperative day, an US scan was repeated, revealing biliary ducts of normal diameter; however, there was an intrahepatic fluid collection described at the point of liver laceration measuring approximately 6 × 2 × 3 cm in size. Exact determination of whether the fluid was hematoma, biliary fluid or other was impossible on US, so we decided to perform cholangiography imaging through the T-tube to exclude biliary leakage. The cholangiography revealed evident leakage from the left hepatic duct, with the size and location of the extravasated contrast corresponding to the previously described fluid collection on US. Furthermore, a small leakage of contrast was seen in the right biliary tree with small collection of contrast with diameter around 5 mm. The common hepatic duct and common bile duct were otherwise intact and draining of contrast media to the duodenum was observed (Fig. 2). A decision was made to manage the leakage of bile from left and right biliary tree conservatively. The T-tube was consequently left open.

**Fig. 3:**
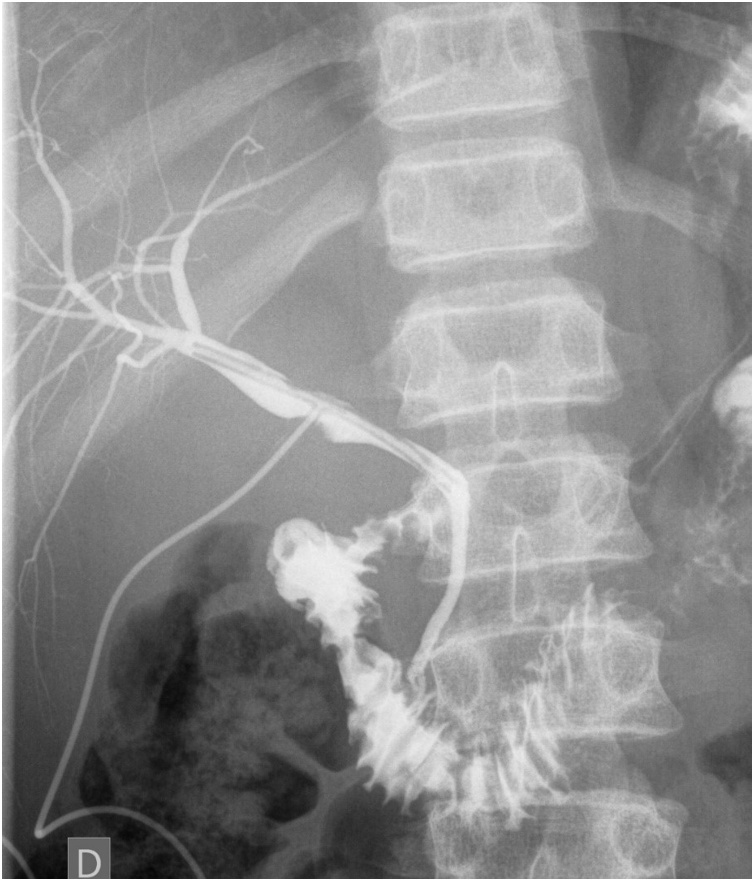
**The cholangiography performed on 18th postoperative day**. Normal opacification of the intra and extra-hepatic biliary channels are noted without signs of biliary leakage. Normal opacification of the duodenum is seen as well.

On the eighteenth postoperative day and 13 days after the first cholangiography, a follow-up cholangiography was performed, which showed no signs of leakage from both the left and right biliary trees (Fig. 3).

In response to the cessation of leakage, we decided to commence intermittent closure of the T-tube. A series of follow-up abdominal US scans were performed, and the size of the intrahepatic collection was initially noted to be increasing, with the largest diameter being 8.6 × 7.1 × 7.6 cm (approximately 300 mL) on the twenty-first postoperative day - 3 days after initiation of the intermittent T-tube closing. Afterwards, the intrahepatic fluid collection began to decrease in size and finally stabilized.

**Fig. 4:**
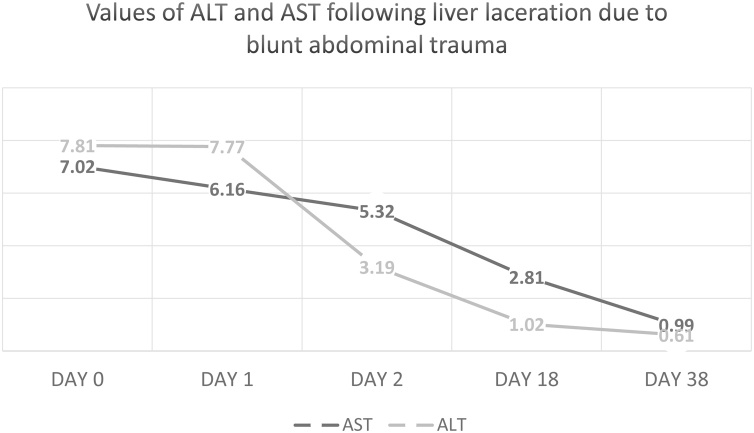
**ALT and ALS levels following liver laceration**. In the course of the inpatient hospital treatments, the values of liver enzymes ALT and ALS decreased to normal levels.

The last US follow-up during hospital stay was performed on the 38th postoperative day and showed a collection measuring 10.6 × 7.4 × 5.9 cm (220–250 ml), indicating that the size of the collection did not change and there were no signs of biliary obstruction. The patient was asymptomatic and feeling well, and we noted a decline of the previously elevated liver enzymes (ALT and ALT) (Fig. 4). Consequently, he was discharged from the hospital with a closed T-tube in place.

## 3 Follow-up and outcomes

The first follow-up in the outpatient clinic was on the 46th postoperative day. The patient was still asymptomatic and feeling well. A check abdominal US scan showed no significant dynamics of the intrahepatic fluid collection (size:10.6 × 7.2 × 5.1 cm, 200–220 ml), and no signs of biliary obstruction. We therefore, decided to remove the T-tube. Due to the normal laboratory results and absence of subjective complaints from the patient, we did not deem it necessary to perform percutaneous drainage of the collection.

Almost one year after the operation, the size of the collection is decreasing, measuring 9.7 × 8.1 × 2.7 cm (approximately 80 mL) and the patient remains asymptomatic.

## 4 Discussion

The bicycle handlebar injury is somewhat unique regarding its location and the need for intervention, compared to other bicycle injuries [3]. An analysis of HBI sites noted that the abdomen is the most frequently injured body region (64.0%), followed by other sites including the face (13.6%), chest (12.3%) and the thigh (11.4%) [7]. Despite minimal or absent visible external signs on the abdominal wall, bicycle handlebar injuries should be treated with great care [2,[6], [7], [8]].

Best practice guidelines recommend that the vast majority of blunt liver trauma in children should be managed conservatively [10,11]. Nevertheless, the guidelines also affirm that hemodynamic status, rather than the grade of the injury, should determine the primary treatment strategy [10,12]. If there is no response after initial fluid resuscitation or there is a great amount of free intraperitoneal fluid with signs of active bleeding on FAST, other imaging techniques should be omitted and an emergency operative protocol activated [10,13]. Compared to other bicycle trauma cases, HBI patients are much more likely to require a major operation [7]. Published case series report a 20–40% operative intervention rate in paediatric HBI patients [4].

In the present report, we describe a case of HBI causing severe liver laceration with haemorrhage and left hepatic duct injury.

Treatment of liver injuries in children can represent an arduous challenge to the paediatric surgeon. Control of haemorrhage is critical but can be difficult to achieve. Several measures to achieve surgical haemostasis have been described in the literature [[12], [13], [14]].

Management of injury to the intrahepatic biliary tract should follow the control of associated haemorrhage [15]. In adults, biliary injuries are commonly iatrogenic (laparoscopic cholecystectomy being the most frequent cause) and there is a large volume of published studies describing the management of such injuries [[16], [17], [18]]. In contrast, there is a limited literature on the management of paediatric biliary tract injury following trauma [11,15].

Traditionally, laparotomy and hepaticojejunostomy has been the gold standard for treatment of biliary injuries [19,20]. Nevertheless, we believe that in a case of a major transection of the intrahepatic biliary duct, primary suture and duct-to-duct anastomosis is feasible if an immediate laparotomy is mandatory due to hemodynamic reasons. It is of vital importance to arrest haemorrhage and attain haemodynamic stability prior to attempting ductal anastomosis. According to literature severe liver lacerations represent a relatively high risk for the development of acute acalculous cholecystitis, thus we decided to perform cholecystectomy. The T-tube drain is useful for biliary drainage and also for later radiographic evaluation if necessary. Possible alternatives to a T-tube drain would be a nasobiliary drain inserted with an ERCP or a reconstruction without a biliary drain. Nevertheless, nasobiliary drain insertion requires an additional procedure which is not without complication and it can also be unsuccessful. A prolonged naso-biliary drainage is far more uncomfortable for the patient as a T-tube. Peritoneal drainage is also advisable in cases of biliary leakage. Additionally, conservative measures such as placement of endoscopic stents to facilitate healing can be carried out if there is no reduction of biliary secretion through the abdominal drain. However, we are aware the bile duct strictures are possible complication after primary suture, but the exact incidence remains unknow due to the lack of scientific data regarding such injuries.

## 5 Conclusion

Due to limited evidence, we recommend consultation with an experienced HPB surgeon on a case-by-case basis for every paediatric biliary injury.

## Timeline of case report

**Figure:**
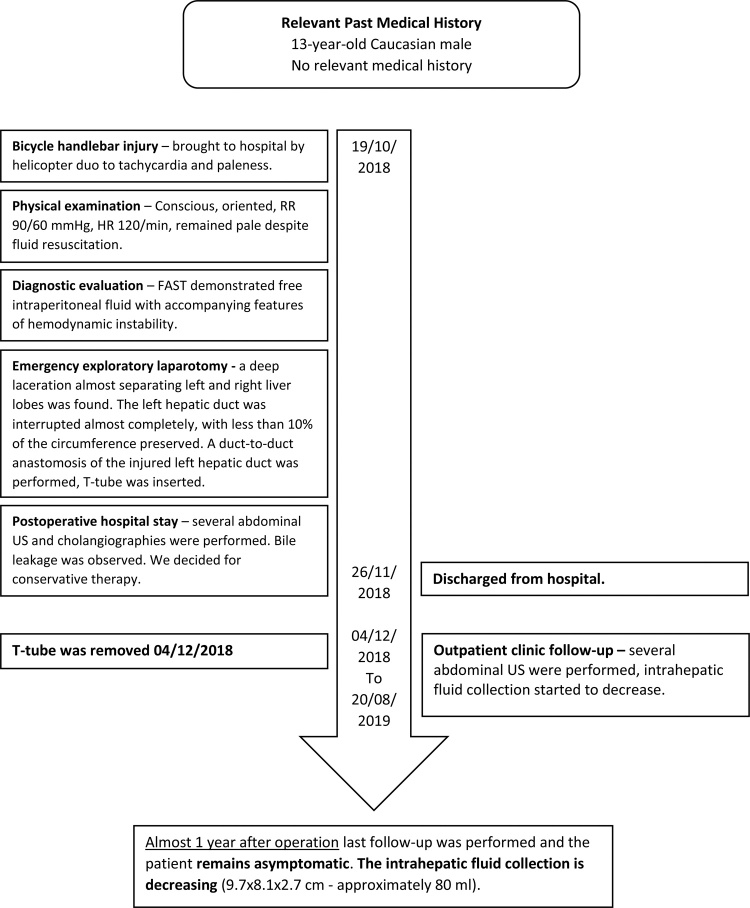

## Declaration of Competing Interest

The authors declare no conflict of interests with regard to this case report.

## Sources of funding

There is no relevant external funding in respect of this case report.

## Ethical approval

Our institution does not require ethical approval for publishing a case report. Written informed consent was obtained from the parents of the young patient for publication of this case report and accompanying images.

## Consent

Written informed consent was obtained from the patient’s parents for publication of this article and any accompanying images.

## Author contribution

Grosek J participated in the operation and drafted the manuscript; Čebron Ž collected the data and was involved in editing the manuscript; Janež J participated in the operation and was involved in editing the manuscript; Tomažič A operated the patient and contributed to critical revision. All authors issued final approval for the version to be submitted.

## Registration of research studies

NA.

## Guarantor

Jan Grosek, MD, PhD.

## Provenance and peer review

Not commissioned, externally peer-reviewed.
